# Proteostasis of organelles in aging and disease

**DOI:** 10.1111/febs.70439

**Published:** 2026-02-04

**Authors:** Yara Nabawi, Cansu Doğan, Dunja Petrović, Gülce Perçin, Seda Koyuncu, David Vilchez

**Affiliations:** ^1^ Institute for Integrated Stress Response Signaling, Faculty of Medicine University Hospital Cologne Cologne Germany; ^2^ Cologne Excellence Cluster for Cellular Stress Responses in Aging‐Associated Diseases (CECAD) University of Cologne Cologne Germany; ^3^ Institute for Genetics University of Cologne Cologne Germany; ^4^ Center for Molecular Medicine Cologne (CMMC) University of Cologne Cologne Germany

**Keywords:** aging, cellular stress responses, membrane‐bound organelles, membraneless organelles, neurodegenerative diseases, organelle dysfunction, protein aggregation, proteostasis, stress granules

## Abstract

To maintain proteome integrity within distinct subcellular compartments, cells rely on tightly regulated proteostasis mechanisms, including protein synthesis, folding, trafficking, and degradation. Disruption of these processes leads to the accumulation of damaged proteins and structural changes that progressively compromise organelle function, contributing to aging and age‐associated disorders, such as neurodegeneration, cancer, and metabolic dysfunction. Here, we discuss recent insights into how proteostasis influences the integrity and function of specific organelles, including the nucleus, mitochondria, endoplasmic reticulum, Golgi apparatus, and lysosomes, as well as membraneless organelles, such as stress granules, processing bodies, the nucleolus, and nuclear speckles. We further discuss how dysfunction in these systems contributes to different hallmarks of aging and disease progression, highlighting potential therapeutic strategies aimed at maintaining organelle homeostasis to promote healthy aging.

## Graphical abstract

Cells rely on regulated proteostasis mechanisms to keep their internal compartments functioning properly. When these mechanisms fail, damaged proteins accumulate, disrupting organelles, such as the nucleus, mitochondria, endoplasmic reticulum, Golgi, and lysosomes, as well as membraneless organelles, such as stress granules, processing bodies, the nucleolus, and nuclear speckles. These changes contribute to aging and age‐related diseases. Understanding how proteostasis preserves organelle function and integrity may reveal strategies to promote healthy aging.

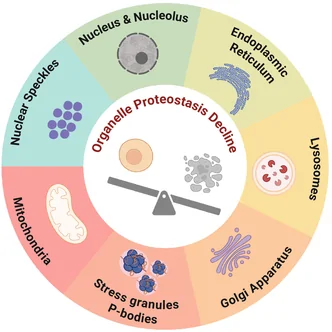

**Abbreviations**
AD: Alzheimer's disease
ALS: Amyotrophic lateral sclerosis
Aβ: Amyloid‐beta
DDR: DNA damage response
ER: Endoplasmic reticulum
FTD: Frontotemporal dementia
Golgi: Golgi apparatus
HD: Huntington's disease
HGPS: Hutchinson–Gilford Progeria Syndrome
Hsp: Heat shock protein
HTT: Huntingtin
IMS: Intermembrane space
ISR: Integrated stress response
LLPS: Liquid–liquid phase separation
mtDNA: Mitochondrial DNA
mtUPR: Mitochondrial unfolded protein response
NDs: Neurodegenerative diseases
NPCs: Nuclear pore complexes
NSs: Nuclear speckles
PBs: Processing bodies
PD: Parkinson's disease
RBPs: RNA‐binding proteins
SASP: Senescence‐associated secretory phenotype
SGs: Stress granules
TIM: Translocase of inner membrane
TOM: Translocate of the Outer (Mitochondrial) Membrane
UPR: Unfolded protein response
UPS: Ubiquitin‐proteasome system

## Introduction

Aging is a complex, multifactorial process characterized by progressive physiological decline. It is one of the greatest risk factors for multiple human diseases, including metabolic syndromes, cancer, and neurodegenerative disorders [1]. Besides late onset, the accumulation of protein aggregates is a hallmark of various neurodegenerative diseases (NDs), including amyotrophic lateral sclerosis (ALS), Alzheimer's disease (AD), Huntington's disease (HD), and Parkinson's disease (PD) [2]. Indeed, pathways regulating proteome integrity, such as protein synthesis, folding, and degradation, are key determinants of longevity and influence the onset and progression of age‐related diseases [1, 3, 4, 5].

Over the course of evolution, organelles have specialized to compartmentalize diverse biochemical reactions and functions, thereby supporting cellular homeostasis. Membrane‐bound organelles, such as the nucleus, mitochondria, endoplasmic reticulum (ER), Golgi apparatus (Golgi), and lysosomes, are central hubs for metabolism, signaling, and protein homeostasis (proteostasis) [6]. In addition, cells contain membraneless organelles, such as nuclear speckles (NSs), stress granules (SGs), and processing bodies (PBs), which are dynamic compartments involved in RNA metabolism and stress responses relevant to proteostasis and aging [7].

**Figure 1:**
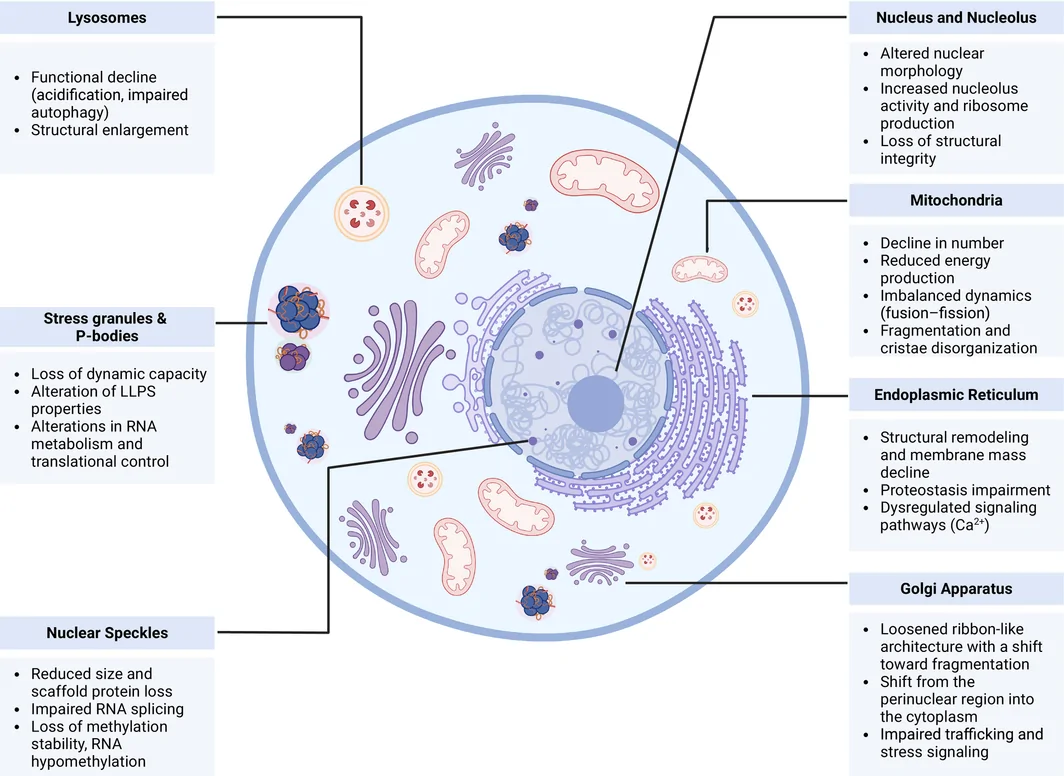
Age‐associated structural and functional alterations in organelles. Aging causes alterations across cellular organelles, including nuclear and nucleolar enlargement, reduced mitochondrial function and number, endoplasmic reticulum remodeling and stress, Golgi apparatus changes, loss of stress granule (SG) and processing‐body (PB) dynamics, and disrupted nuclear speckle organization.

As organisms age, the composition, morphology, and function of organelles become progressively compromised (Fig. 1). This age‐associated organelle dysfunction contributes to the accumulation of cellular damage, impaired proteome integrity, defective stress responses, and the onset of age‐related diseases [8, 9, 10, 11]. In this review, we discuss recent advances in understanding the interplay between organelle homeostasis, proteostasis, and aging.

## Membrane‐bound organelles

Organelles such as the nucleus, mitochondria, ER, Golgi, and lysosomes are surrounded by lipid membranes. The compartmentalization of these organelles creates specialized environments that require organelle‐specific proteostasis mechanisms. Thus, these organelles rely on intrinsic protein quality control systems and stress responses tailored to their unique microenvironments and functions [6, 12, 13]. In the following sections, we discuss how aging and disease alter proteostasis in different membrane‐bound organelles and how these deficits contribute to other age‐related and pathological changes.

### Nucleus

The cell nucleus is the largest and one of the most highly structured organelles, composed of various substructures. This organelle houses the genome and facilitates transcription as well as various RNA‐processing steps. Maintaining nuclear architecture, proteome integrity, and function is essential for normal cell activity and organismal health. Nuclear proteostasis refers to the specialized protein quality control systems operating within the nucleus and its various subcompartments. This process primarily involves nuclear‐localized molecular chaperones, the ubiquitin‐proteasome system (UPS), and autophagy mechanisms, such as nucleophagy. Together, these components work to maintain proteostasis by ensuring proper folding and degradation of nuclear proteins [14]. The nucleus is closely linked to several hallmarks of aging beyond proteostasis loss, including telomere attrition and genomic instability [1]. Conversely, abnormalities in nuclear properties, such as nuclear genome instability or alterations in nuclear morphology and architecture, disruption of nuclear‐cytoplasmic trafficking, and impaired ribosome biogenesis (see the section Nucleolus) are common features of aging and cellular senescence [15, 16, 17, 18, 19].

The nucleus is enclosed by a double membrane called the nuclear envelope, which contains three key components: the nuclear membranes, nuclear pore complexes (NPC), and nuclear lamina [20]. These components form specialized environments necessary for dedicated proteostasis mechanisms and maintenance of nuclear proteome integrity. Alterations in nuclear envelope components contribute to morphological changes during aging, including enlargement and loss of structural integrity. For example, human gingival fibroblasts from older individuals exhibit larger, irregularly shaped nuclei compared with those from younger individuals [21]. Similarly, cortical neurons from aged mice show increased nuclear size compared with neurons from young mice [22]. In *Caenorhabditis elegans*, age‐related alterations to nuclear architecture, including deterioration in nuclear shape and heterochromatin loss, are observed in most non‐neuronal tissues. Conversely, longevity paradigms such as reduced insulin/IGF‐1‐like signaling can ameliorate these changes [23].

Altered nuclear morphology often accompanies changes in the nuclear lamina, a protein matrix of intermediate filaments primarily responsible for nuclear stiffness and nucleocytoskeletal coupling. The nuclear lamina is mainly composed of lamins A/C and B [24]. Functionally, it is particularly important in mechanically active tissues, such as muscle, playing a key role in proper nucleus positioning [24]. Growing evidence indicates that altered lamin B1 expression is closely associated with aging and age‐related pathologies. For instance, human fibroblasts and keratinocytes show a marked decline in lamin B1 levels during replicative senescence [25, 26]. The role of lamin B1 in aging is further supported by *in vivo* studies. In *Drosophila melanogaster*, the lamin B1 ortholog LAM declines in fat body and brain with age, although not in the gut or heart tissues [27]. In *C. elegans*, postembryonic knockdown of the lamin ortholog *lmn‐1* reduces lifespan, indicating that lamins are critical for longevity [23]. Mutations in lamin A/C are associated with premature aging syndromes, such as Hutchinson–Gilford Progeria Syndrome (HGPS). In this disorder, the accumulation of mutant lamin A/C protein, known as progerin, causes structural abnormalities in the nucleus. This not only distorts nuclear‐cytoplasmic transport but also leads to premature cellular senescence. Consequently, these changes contribute to increased cell death and accelerate organismal aging. Furthermore, human fibroblasts derived from HGPS patients also exhibit lamin B1 reduction [28].

Nuclear proteome maintenance heavily depends on nuclear‐cytoplasmic transport, and NPCs play a central role in this cellular function [29, 30, 31, 32]. Proteostasis imbalance due to NPC deterioration results in the accumulation of misfolded and damaged nuclear proteins, accelerating aggregate formation and cellular dysfunction observed in aging and age‐related diseases [17, 33, 34, 35, 36]. For instance, in postmitotic cells, structural depreciation of NPCs, including loss of NUP complex proteins like Nup93, leads to increased nuclear permeability, which in turn exacerbates proteostasis collapse during aging [37, 38]. In aged yeast, the abundance of NPC components and assembly factors declines, thereby impairing transcription factor shuttling and increasing nuclear compartmentalization [35]. Moreover, disruptions in nucleocytoplasmic transport are highly linked to age‐related NDs, including HD and ALS [36, 39, 40]. Studies using iPSC‐derived neurons from ALS patients and model organisms show that expression of ALS‐associated mutant C9orf72 protein impairs NPC localization and nuclear import, contributing to pathological changes [36, 39].

One of the main components of the nucleus is DNA, which is organized into chromatin, a complex of DNA and proteins. DNA repair capacity declines with age, and the resulting accumulation of DNA damage is a hallmark of aging and age‐related diseases [1, 41]. The buildup of DNA lesions activates a range of signaling pathways, known as the DNA damage response (DDR). Chronic DNA damage can alter the epigenome, impair DNA replication and transcription, and trigger cellular senescence or apoptosis [42]. DNA damage markers are frequently observed in age‐related diseases, such as cancer, neurodegenerative disorders, and cardiovascular disorders [43]. Defective DNA repair can also drive premature aging in multiple organs. For example, mutations affecting global genome nucleotide excision repair increase susceptibility to skin cancer and may also promote neurodegeneration [44, 45, 46, 47, 48]. Particularly, mutations in RecQ helicase, which regulates telomere stability and replication stress, cause progeroid syndromes, such as Werner and Bloom syndromes [49]. In *C. elegans*, persistent DNA damage in DDR‐deficient animals induces a proteostasis shift toward autophagy‐mediated proteolysis as an adaptive mechanism to cope with genotoxic stress [50].

Proteostasis mechanisms are critical for supporting DNA repair by regulating the synthesis, stability, and turnover of repair factors. Their decline impairs genome maintenance and leads to increased cellular senescence and cell death [42, 51, 52]. The conformational stability of many DNA repair proteins depends on molecular chaperones [53, 54, 55, 56]. One well‐studied example is heat shock protein 90 (HSP90), which plays a critical role in many repair processes. Inhibition of HSP90 sensitizes human cells to UV exposure, and HSP90 has been reported to accumulate at DNA damage sites [57, 58].

Proteolytic pathways, including autophagy and the UPS, which become dysregulated with aging, also contribute to the regulation of genome integrity [1, 59, 60]. Particularly, autophagy plays a key role in genome integrity by selectively degrading nuclear components through nucleophagy [61]. For example, basal nucleophagy contributes to genome stability by degradation of type II topoisomerases A and B as well as nucleolar components [62]. Impairment of autophagy can directly enhance DNA damage, as deletion of Atg7 in murine keratinocytes results in severe DNA damage and cellular senescence [52].

Moreover, chromatin organization is tightly linked to proteostasis, as histone turnover, remodeling complex activity, and the stability of epigenetic enzymes all depend on protein quality control mechanisms. Molecular chaperones facilitate histone folding and exchange, supporting the dynamic regulation of chromatin accessibility and epigenetic states, whose alterations are associated with aging [1, 63, 64, 65]. For instance, the molecular chaperone HSP90 interacts with Trithorax, a member of a chromatin protein complex that modulates epigenetic signaling, highlighting a direct intersection between proteostasis and chromatin regulation [65]. In addition to chaperones, proteolytic systems play a critical role in maintaining chromatin homeostasis. Impaired proteasome activity can alter nucleosome occupancy and histone modification patterns, thereby reshaping the chromatin landscape and influencing transcriptional programs [66, 67, 68]. Beyond proteasome‐mediated degradation, autophagy also contributes to chromatin remodeling. Emerging evidence indicates that autophagy modulates epigenetic regulation by selectively degrading chromatin‐associated proteins [69, 70]. Thus, nuclear proteostasis preserves chromatin structural integrity and ensures the fidelity of epigenetic regulation, whose progressive deregulation is a key driver of cellular aging.

Progressive telomere shortening contributes not only to DNA damage and replicative senescence during aging, but also challenges nuclear proteostasis [71, 72, 73, 74]. Once telomeres become critically short, they fail to bind sufficient telomere‐covering proteins, leaving DNA ends exposed. This triggers a DDR and inhibits cell proliferation, ultimately leading to senescence. Accumulated senescent cells release pro‐inflammatory cytokines, a phenomenon referred to as the senescence‐associated secretory phenotype (SASP). The SASP not only modulates extracellular matrix composition but also propagates senescence to neighboring cells, contributing to chronic inflammation [75]. Thus, telomere attrition‐associated DDR has been proposed as a driver of organismal aging [72, 76].

Taken together, compromised nuclear architecture and genome instability, which are tightly linked to proteostasis mechanisms, are central determinants of aging. Age‐related changes in nuclear function, morphology, and lamina composition contribute to aging phenotypes, such as cellular senescence, chronic inflammation, and impaired proteostasis. Further elucidating the mechanisms underlying these changes will be critical for advancing our understanding of the biology of aging and longevity.

### Mitochondria

Mitochondria, originating from ancient alphaproteobacteria through symbiosis, are more than just the ‘powerhouses’ of the cell. They are involved in essential biological processes, such as cell signaling, lipid synthesis, calcium and redox homeostasis, cell division and cell death [77]. In fact, their semi‐autonomous nature, harboring mitochondrial DNA (mtDNA), ribosomes—generally termed as mitoribosomes—and tRNA, reflects their unique protein synthesis machinery but also makes them vulnerable to mtDNA mutations. Impaired mitochondrial function disrupts cellular proteostasis, ultimately compromising organismal health.

Mitochondrial homeostasis dysfunction, such as decreased mitochondrial function and stress resilience, can serve as a biomarker of aging. Therefore, understanding mitochondrial structure and distribution patterns is essential for interpreting their roles in aging and age‐associated diseases. Cells with high energy demands, such as cardiomyocytes and skeletal muscle cells, exhibit increased numbers and spatial distributions of mitochondria compared with hepatocytes of the same organism [78]. Neurons, the longest cells in the human body, require enhanced energy production at specific intracellular locations; therefore, mitochondria display a distinct distribution and behavior, suggesting that intracellular micro‐niches play a critical role in dictating mitochondrial function [79]. With age, these finely tuned spatial arrangements and numbers are altered, leading to both structural and functional changes. Mitochondrial numbers are significantly decreased independent of tissue types. However, the rate of decline in mitochondrial numbers and function remains tissue‐specific [80]. Defining the mitochondrial number and subcellular localization may serve as a valuable biomarker for predicting tissue age and age‐mediated disease progression.

Structurally, mitochondria are double‐membrane organelles, consisting of an outer and a highly folded inner membrane, where the electron transport chain resides. The mitochondrial matrix contains mtDNA, mitoribosomes, and enzymes of the tricarboxylic acid cycle. Sustained energy production depends on functional mitochondrial proteostasis, which requires efficient import, stabilization, folding, and quality control of precursor polypeptides transported from the cytoplasm. Mitochondrial proteostasis is maintained by a coordinated network of mitochondrial resident proteins that ensure correct protein import and folding, and when necessary, degradation for preventing aggregation. As the vast majority of mitochondria resident proteins are synthesized in the cytosol, translocases and chaperone proteins are central to these synthesis processes, facilitating protein transport, folding, and assembly while preventing aggregation within distinct mitochondrial locations.

Protein import into mitochondria is mediated by Translocate of the Outer Mitochondrial Membrane (TOM) complexes, the main entry gate of the mitochondria. TOM complexes are major translocases that cooperate with cytosolic chaperones to allow selective entry of precursor proteins into mitochondria. Precursor proteins synthesized in the cytosol are recognized by TOM receptors, such as Tom20 and Tom22 [81], and translocated across the outer membrane through the Tom40 channel into the intermembrane space (IMS) with the assistance of IMS‐specific translocase of inner membrane (TIM) chaperones [82].

Unlike matrix chaperones, IMS chaperones function in an ATP‐independent manner [83] and form a part of the small TIM complexes, such as TIM9–10 and TIM8–13. These ‘holdase’ chaperones escort hydrophobic precursor proteins across the IMS, maintaining their conformation for insertion into the mitochondrial inner membrane while preventing aggregation [84]. Thus, large TIM complexes, primarily TIM22 and TIM23, are also critical for mitochondrial proteostasis. The TIM23 complex defines the number of mitochondrial translocation contact sites and sorts precursor proteins to mitochondria‐specific locations, acting as an essential translocase [85]. The TIM22 complex inserts multi‐spanning membrane proteins, such as metabolite carriers, into the inner mitochondrial membrane through interactions with IMS‐localized small TIM chaperones, thereby maintaining mitochondrial health and function [86]. Together, these interactions highlight the sophisticated chaperone‐mediated transport of precursor proteins within the mitochondria, forming the backbone of mitochondrial proteostasis.

Matrix chaperones play a central role in folding and stabilizing proteins. mtHsp70 mediates the import of mitochondrial preproteins [87] and, together with the co‐chaperones (Mdj1 and Mge1), regulates their folding, stabilization, and refolding [88, 89]. All these activities depend on ATP‐mediated mtHsp70 function, preventing the accumulation of short hydrophobic peptides [90]. Consistently, mtHsp60, together with its co‐chaperonin mtHsp10, facilitates the folding of mitochondrial matrix proteins [91], and regulates the refolding of damaged proteins under chemical or heat stress [92]. mtHsp78, another mitochondrial matrix chaperone, is essential for protein refolding under heat stress and functions in collaboration with mtHsp70 [93]. When refolding fails, these chaperones target misfolded proteins to proteases for degradation to prevent protein aggregation. Although mtHsp70 and mtHsp60 often act in concert [91], they can also operate independently, highlighting the substrate‐specific nature of chaperone‐mediated folding within the matrix. TRAP‐1, an Hsp90 chaperone family member in the mitochondria matrix, regulates the metabolic balance between oxidative phosphorylation and aerobic glycolysis [94], adding another layer to the classical chaperone functions. Together, these chaperones are not only essential for protein import, folding, and stabilization, but also for maintaining mitochondrial quality control by ensuring degradation of misfolded proteins via mitochondrial proteases.

Specific mitochondrial proteases reside in distinct compartments to maintain proteome integrity, regulate fusion and fission, cleave precursor proteins, and activate the mitochondrial unfolded protein response (mtUPR) as a last resort for mitochondrial protein quality control mechanism. In the matrix, LON and ClpXP proteases as well as mitochondrial processing peptidases and mitochondrial intermediate peptidases maintain proteostasis within this organelle. Membrane‐anchored, ATP‐dependent proteases, including m‐AAA [95], i‐AAA, Oma1, and Ymel1, are located in the inner membrane, whereas HtrA2, also known as Omi protease [96], resides in the IMS. This compartmentalized organization highlights the spatiotemporal complexity of proteases' specificity required for mitochondrial proteostasis, with distinct chaperones directing misfolded proteins to their targeted proteases. For example, in mammals, mtHsp70 and mtHsp60 direct misfolded proteins to the LON protease within the mitochondrial matrix, contributing to proteostasis. In yeast, the mitochondrial chaperone Hsp78 (a ClpB homolog, not present in mammals) cooperates with ClpXP in disaggregating and refolding proteins under stress conditions, helping to prevent protein aggregation and maintain mitochondrial function [97]. Nevertheless, a complete atlas of mitochondrial chaperone–protease interactions remains to be achieved.

The accumulation of misfolded or unfolded proteins exceeding the capacity of mitochondrial chaperones and proteases leads to mitochondrial stress, activating the mtUPR [98]. Initiated in the mitochondrial matrix or inner membrane, this stress triggers proteotoxicity characterized by elevated mitochondrial ROS production [99] and defective protein import, which in turn leads to cytosolic buildup of mitochondrial‐targeted proteins and subsequent activation of the mtUPR [99, 100]. In the mtUPR, oxidation of DNAJA1, a co‐chaperone of cytosolic Hsp40, recruits cytosolic Hsp70, which further promotes the activation of heat shock factor 1 (HSF1) transcription factor. Activated HSF1 then induces the transcription of mitochondrial chaperones, such as mtHsp60, mtHsp78 (in yeast), and co‐chaperones Mdj1 and Mge1 [99]. In parallel, ATF5, another transcription factor, also contributes to mtUPR by upregulating mitochondrial chaperones mtHsp60 and LON protease. Additionally, alterations in the mtUPR can further engage the integrated stress response (ISR) in mammals by phosphorylating eIF2a, leading to ATF4 translation and subsequent ATF5 expression [101, 102]. This ISR‐linked program promotes the expression of mitochondrial chaperones and proteases, thereby reinforcing mitochondrial proteostasis as an adaptive protective mechanism. Together, these observations suggest that, although safeguarding mitochondrial integrity is evolutionarily conserved, the signaling pathways coordinating mitochondrial stress responses have diverged across species. However, the precise mechanisms of mtUPR‐mediated transcriptional regulation, including how specific transcription factors regulate distinct subsets of mitochondrial chaperone and protease gene transcription, remain incompletely understood.

Mitochondrial peptidases are another quality control layer which is significantly involved in proteostasis in the context of aging and age‐related NDs. Pitrilysin metallopeptidase 1 (PITRM1) is a mitochondrial matrix peptidase involved in the digestion of mitochondrial‐targeting sequences of proteins that are imported across the IMM [103]. Defects in PITRM1, or its loss, are associated with amyloid‐beta (Aβ) accumulation within the mitochondria, as shown in yeast and mouse models [104], as well as AD‐like pathology in human cerebral organoids [105]. Another peptidase involved in proteostasis maintenance within the mitochondria is the metalloprotease PreP. This enzyme is responsible for degrading the mitochondrial‐targeting sequence of specific proteins into small fragments after initial cleavage by the mitochondrial processing peptidase [106]. Oligopeptidases are then responsible for further degrading the cleaved signaling peptides into amino acids. All of these sequential processing mechanisms are essential for proper activity and localization of mitochondrial proteins. Furthermore, defects in these cleavage pathways are highly associated with aggregate formation, activation of the mitochondrial ISR, and loss of proteostasis [107, 108]. Together, these complex pathways ensure mitochondrial proteostasis. Disruption at any level (i.e., protein import, chaperone function, or proteolysis) results in proteotoxic stress and metabolic imbalance, hallmarks of aging and degenerative disease.

During aging, the functionality of mitochondrial import translocases TOM and TIM complexes, mitochondrial chaperones, proteases and the mtUPR progressively declines. Alterations in mitochondrial protein import or loss of mitochondrial peptidase expression and function are thought to be key drivers of aging and age‐associated diseases. In fact, dysfunctions within the mitochondrial protein import machinery, particularly the TOM and TIM complexes, represent some of the earliest and most sensitive biomarkers of mitochondrial aging [109]. The activating transcription factor associated with stress 1 (ATFS‐1) is an essential component for regulating the mtUPR. In *C. elegans*, ATFS‐1 has been linked to promoting the accumulation of deleterious mtDNA, which is associated with aging [110].

The expression of TOM70 and TOM20, components of the TOM complex, significantly declines in the hippocampus and cortex of aged mice and in AD patients, predominantly compromising mitochondrial proteostasis by reducing the import of essential proteins into mitochondria [111]. While age‐mediated loss of TOM70 accelerates mitochondrial aging and age‐dependent mitochondrial dysfunction, overexpression of TOM70 delays these mitochondrial dysfunctions and extends lifespan in budding yeast [112]. TOM40, the central pore‐forming subunit of the TOM complex, is a direct indicator of neuronal health. Homozygous *Tom40*+/− mice exhibit heart impairments and peripheral neuropathy, resulting in earlier death compared with littermate controls. In addition, single nucleotide polymorphisms within *TOMM40s* are associated with late‐onset AD [113]. Declined TOM40 levels or dysfunction of the TOM complex resulting from TOM40 loss are associated with both AD and PD [114]. Together, impaired mitochondrial import represents an early event linking mitochondrial homeostasis directly to aging and age‐related NDs.

Age‐related, tissue‐specific changes are further reflected in those chaperones required for maintaining mitochondrial quality control by facilitating the degradation of misfolded proteins via mitochondrial proteases. Within the IMS, although TOM protein expression declines with aging, TIM proteins—particularly TIM23—appear to compensate for this decline, supporting neuronal function and health in humans [115]. TIM23 heterozygous knockout mice display neurological disease phenotypes and reduced lifespan, further suggesting that the relative increase of TIM23 in aging neurons may act as a compensatory mechanism. Furthermore, postmortem PD brain samples show decreased TIM23 expression, highlighting the importance of functional TIM23 in preventing age‐related mitochondrial alterations [115].

In addition, the essential matrix chaperone mtHsp70 declines in both expression and function in neuronal tissues during aging and has been linked to age‐related neurodegeneration due to the accumulation of misfolded proteins [116]. Consistently, overexpression of mtHsp70 in murine skeletal muscle protects against muscle damage and age‐mediated muscle dysfunction [117]. Notably, long‐living organisms exhibit elevated levels of mtHsp70 [118], suggesting that mtHsp70 functions as a protective factor against aging and age‐related diseases. Similarly, point mutations in mtHsp60 lead to severe hereditary spastic paraplegias, causing progressive muscle spasticity and lower‐limb weakness [119]. Taken together, declines in chaperone expression and function disrupt mitochondrial proteostasis, resulting in age‐related mitochondrial stress and degeneration.

### Endoplasmic reticulum

The ER is a continuous, single‐membrane multifunctional organelle essential for maintaining cellular proteostasis via protein synthesis, folding, and quality control. In addition, it regulates lipid and glucose metabolism, calcium signaling, and cellular stress responses. The ER serves as a nexus of interorganelle interaction, physically surrounding and functionally interacting with nearly every organelle and the plasma membrane, thereby directly impacting proteostasis [120].

The ER is structurally dynamic and includes specialized subdomains, such as the nuclear envelope and peripheral networks of sheets and tubules. ER comprises two major structural domains: the rough ER, with ribosome‐studded membranes for protein synthesis, and the smooth ER, a network of ribosome‐free tubules involved in lipid metabolism, detoxification, and calcium homeostasis. ER domains undergo structural remodeling in response to cellular demands by reshaping sheets and tubules into the rough and smooth ER architecture [121, 122].

Growing evidence indicates that ER structure and function are compromised with age [11, 123, 124, 125]. For instance, young neurons display highly ordered parallel cisternae of rough ER, which become progressively more dispersed in both aged mitral cells in the olfactory bulb and in Purkinje cells [126]. During aging, ER morphology shifts from organized rough ER sheets densely packed with ribosomes to a more diffuse tubular network, suggesting a decline in protein synthesis capacity and ER functional integrity [127]. As the ER is a key compartment for protein folding and modification, its structural changes during aging impair proteostasis. The accompanying large‐scale shifts in proteomic composition suggest a functional shift in ER from proteostasis to lipid processing with age [127, 128].

Chronic accumulation of misfolded peptides triggers ER stress and activates all three branches of the unfolded protein response (UPR), leading to cell death. These three ER‐resident transmembrane sensors are protein kinase R (PKR)‐like endoplasmic reticulum kinase (PERK), inositol‐requiring enzyme 1 (IRE1), and transcription factor 6 (ATF6) [129, 130]. During aging, the ER‐resident transmembrane sensors become desensitized, leading to decreased expression of downstream transcriptional targets. Among those, specifically, IRE1 is associated with cellular senescence. IRE1 activation leads to the regulation of the transcription factor X‐box binding protein 1 (XBP1) through its endoribonuclease activity, resulting in the activation of the spliced form of XBP1 (sXBP1), which upregulates the expression of chaperones and regulates ER proteostasis [131]. The constitutively active form of XBP1s in neurons extends longevity and improves UPR(ER) signaling in aged *C. elegans* [128]. Notably, overexpression of XBP1 rescues the senescence phenotype in aged rat brains, improving the IRE1‐dependent UPR, which might suggest an alternative treatment specific for ER‐mediated diseases [132].

On the contrary, PERK expression and eIF2α phosphorylation are markedly reduced in aging rodent brains along with a progressive decline in hippocampal PERK mRNA levels [132]. In *Drosophila*, sleep deprivation is associated with ER stress, as evidenced by reduced sXBP1 levels and elevated eIF2α phosphorylation. Remarkably, experimentally inducing ER stress in young flies restores normal sleep architecture and mitigates sleep fragmentation [126]. The ATF‐6 branch also influences cellular aging by regulating ER calcium homeostasis via age‐dependent upregulation of its transcriptional target, the chaperone calreticulin. Loss of ATF‐6 reduces calreticulin levels, thereby enhancing calcium transfer to mitochondria through IP3R and MCU‐1 channels. This improvement in mitochondrial function ultimately promotes organismal longevity [133].

Notably, increased contact sites between mitochondria and the ER extend lifespan in an age‐associated AD model in *Drosophila* [134]. This further implies that the ER, as the main interorganelle bridge, plays a key role in coordinating cellular proteostasis. In early life, the UPR regulates protective and apoptotic responses to maintain ER homeostasis. With age, this balance shifts toward cellular dysfunction and death. For instance, ER stress induces elevated levels of the pro‐apoptotic protein CHOP, and caspase‐12 expression is significantly higher in the hippocampus and cortex of aged mice. These changes are not confined to the brain but are also observed in the retina, skeletal muscle, and adipose tissue of male mice [135].

There is a significant age‐dependent decline in ER‐resident proteins and chaperones like PDI and BIP, which are required for proper protein folding and degradation of misfolded proteins contributing to maladaptive activation of the UPR [136, 137]. This decline, likely worsened by oxidative damage, compromises protein quality control and promotes toxic protein accumulation [136]. Age‐related loss of ER chaperone capacity and elevated UPR(ER) are associated with ER stress, which contributes to alterations in ER size and the shape of aging cells [138]. In addition to ER stress, there is a pronounced loss of ER protein content and membrane mass with age. This suggests that ER‐phagy, which is the process of selective degradation of ER subdomains, can contribute to ER remodeling during aging. For instance, ER‐phagy reduces protein synthesis capacity by removing rough ER [127, 139]. Notably, calnexin, a chaperone that declines with age, also functions as an ER‐phagy co‐receptor by partnering with FAM134B to recognize misfolded procollagens [126, 140]. Loss of calnexin impairs the recruitment of substrates for degradation. Other chaperones, such as calreticulin, ERp57, and UGT1, are also required for proper ER‐phagy [141].

Interestingly, the UPR induces ER expansion to dilute protein load while increasing chaperone levels. Expansion of the ER following UPR(ER) activation is selectively reversed through a form of autophagy known as RecovER‐phagy, mediated by the translocon component SEC62, underscoring mechanistic differences in ER remodeling during stress recovery. Therefore, ER size reduction during aging may not be solely degenerative but could also reflect adaptive remodeling [142]. Strikingly, long‐lived naked mole rats have evolved the ability to rapidly adapt to challenging conditions by modulating their ER structure, shifting from rough to smooth ER to enhance metabolic flexibility and stress resistance [143]. In long‐lived species, ER remodeling enhances proteostasis, with the nuclear factor erythroid 2‐related factor (Nrf2) proposed as a contributing regulatory transcription factor. Long‐lived animals have high Nrf2 regulation, which sustains ER integrity by upregulating molecular chaperones, stimulating ER‐phagy, and promoting the clearance of misfolded proteins [144, 145, 146]. In addition, ER expansion dilutes unfolded proteins, thereby reducing their toxicity. Collectively, Nrf2 is a potential target for maintaining ER health in the context of aging and disease.

Dietary restriction (DR) delays proteostasis collapse by sustaining ER stress responses and ER quality control mechanisms during adulthood of *C. elegans*, thereby extending their lifespan. This effect is partly due to a sublethal ER stress during early development, which primes the ER for enhanced function later in life [147]. While DR promotes ER remodeling to support healthy aging, a complementary strategy targets ER biogenesis through mTOR signaling. Senescent cells rely on mTOR‐associated expansion of the ER to sustain the high protein synthesis required for the SASP. A recent study showed that inhibiting mTOR signaling via the drug DDIT5 induces ER stress and triggers apoptosis, even in proliferating cells [147]. By manipulating mTOR signaling and remodeling the ER, it may be possible to significantly enhance our ability to delay aging and age‐related diseases.

### Golgi apparatus

Membrane, secreted, and lysosomal proteins synthesized and folded in the ER are subsequently transported to the Golgi, a central organelle that orchestrates processing, chemical modification, and sorting of proteins and lipids before delivery to their final destinations. Besides its role in regulating secretory pathways, the Golgi is involved in cell cycle regulation, microtubule organization, and cell polarization, as well as in the modulation of apoptosis and stress responses [148, 149, 150, 151].

The Golgi also contributes to the maintenance of cellular proteostasis. While the main quality control steps of the secretory pathway occur in the ER, an additional checkpoint localizes to the Golgi. Within the Golgi, oligomerized or aggregated proteins are directed to lysosomal degradation, while misfolded proteins are transported back to the ER [152, 153, 154]. In addition, Golgi membranes serve as scaffolds for the initiation of a conserved, noncanonical autophagy pathway known as Golgi membrane‐associated degradation, which is primarily involved in the degradation of Golgi‐trafficking cargo [155, 156, 157]. The Golgi further plays an important role in the internalization of infectious prion protein species and their delivery to the endolysosomal system [158].

Within vertebrate cells, the Golgi is a polarized organelle dominantly organized into a perinuclear ribbon‐like structure formed by laterally connected stacks of cisternae, which are further supported by the cytoskeleton [148]. The *cis* side of the organelle receives vesicles with ER‐derived cargo, which undergoes maturation before being packed in the vesicles on the *trans* side. Golgi structure is primarily maintained by protein–protein interactions and further stabilized by protein‐RNA condensates [159, 160]. This suggests that age‐induced perturbations in both protein and RNA metabolism might contribute to age‐related changes in Golgi organization.

A tightly regulated and reversible disassembly of the Golgi is an integral part of physiological processes, such as cell division and differentiation [149, 161, 162]. However, loss of Golgi integrity is a common hallmark of cellular stress, pathogen infection, cargo overload, and neurodegeneration [163, 164, 165]. While the role of the Golgi in health and disease has been extensively studied through genetic and pharmacological interventions that trigger Golgi stress response and lead to Golgi dysfunction, the interplay between its function and aging remains elusive. Furthermore, disruption of Golgi function might not only impair cargo modification and trafficking but also exacerbate the accumulation of misfolded proteins and pathological aggregates.

Altered expression of Golgi‐resident proteins contributes to its dispersal in human senescent cells and aged mouse brain [166, 167, 168]. Furthermore, age‐dependent motor neuron degeneration in progeroid DNA repair‐deficient Errc1 mice is accompanied by abnormal Golgi morphology and localization [169]. In the aging bone microenvironment, Golgi disruption along with dysregulated extracellular vesicle release contributes to bone marrow mesenchymal stem cell senescence and decreased bone regeneration capacity [170].

Proper maturation and post‐translational modification of proteins passing through Golgi are highly dependent on its slightly acidic pH, which gradually decreases along the *cis–trans* axis [171, 172]. This pH gradient is regulated by the activity of the vacuolar H + ‐ATPase (v‐ATPase) [173]. Lower expression of the *trans*‐Golgi v‐ATPase subunit ATP6V0A2 has been associated with Golgi fragmentation and altered glycosylation patterns in senescent cells [168]. Furthermore, age‐related zinc deficiency, an essential metal involved in protein–protein interactions required for maintaining Golgi stacking, induces Golgi stress and disrupts its function and morphology in senescent fibroblasts [174]. Both zinc and pH homeostasis are also essential for ERp44‐mediated protein traffic and quality control at the ER‐Golgi interface [175], suggesting that their disbalance can contribute to proteostasis decline during aging.

Golgi morphology and cellular secretion are readily modulated upon extracellular stimuli by G protein‐coupled receptor activation. Translocation of G protein βγ complex to Golgi results in its reversible fragmentation and secretory vesicle formation [176]. However, upregulation of the rapidly translocating γ11 subunit, a hallmark of senescence, promotes persistent Golgi vesiculation [177, 178].

Functional and morphological changes in the Golgi are well‐characterized hallmarks of several age‐dependent NDs, including AD, HD, and PD, as well as ALS [163, 165, 179]. While it is still unclear what role the Golgi plays in neurodegeneration, several studies indicate that the changes in morphology are already visible before any observable clinical dysfunction, suggesting that the Golgi might actively contribute to disease progression rather than presenting a mere pathological consequence [179].

Golgi atrophy and fragmentation in motor neurons have been reported during the early phases of ALS in both mouse models and in patients with sporadic or familial disease [180, 181, 182]. In PD, α‐synuclein prefibrillar aggregates similarly disrupt Golgi architecture and protein secretion [183]. In AD, accumulation of Aβ and hyperphosphorylation of Tau induce Golgi fragmentation through Cdk‐5‐dependent phosphorylation of Golgi tethering proteins, thereby impairing their interactome [184, 185]. The Golgi‐resident protein ACBD3 is upregulated in the brains of HD patients as well as in cellular and mouse models [186]. While Huntingtin (HTT) is a known interacting partner of ACBD3 under physiological conditions, the formation of this complex is one of the main drivers of mutant HTT toxicity in HD [186]. Neurons exhibit a unique Golgi spatial organization, consisting of a characteristic ribbon‐like structure in the soma, and dendritically localized mini‐stacks known as Golgi outposts [187, 188]. However, the accumulation of toxic polyQ‐expanded proteins impairs Golgi outposts synthesis and contributes to dendritic pathology in class IV dendritic arborization (C4da) sensory neurons in HD *Drosophila* models [189]. Golgi dysfunction has also been linked to several cancer types [190].

While Golgi alterations are common features of many pathological conditions, their impact likely varies among different cell types. Neurons, in particular, appear to be especially vulnerable, reflecting both their unique Golgi architecture and the wide spectrum of NDs associated with Golgi atrophy. Moreover, given that senescent cells are characterized by SASP, the Golgi may represent a critical regulatory node and potential therapeutic target for mitigating age‐related secretory and proteostatic imbalances. Future studies will be essential to elucidate the contribution of Golgi alterations to cellular aging and disease progression.

### Lysosomes

Lysosomes are central organelles for cellular proteostasis and quality control, facilitating the degradation of macromolecules and recycling of catabolites. To sustain their degradative role, lysosomes contain a wide range of hydrolases, including proteases, lipases, nucleases, and phosphatases which can function optimally in low pH condition (pH 4.5–5). Beyond degradation, lysosomes also serve as key signaling hubs that regulate cellular metabolism, nutrient sensing, and the integration of energy and growth signals. Through these functions, lysosomes enable cells to adapt to various stresses, including protein aggregation, oxidative stress, infection, and organelle damage [191].

Lysosomal dysfunction is recognized as a hallmark of aging and age‐related diseases [192]. During aging, lysosomes undergo profound alterations, such as increased number and size, pH neutralization, accumulation of undigested content, alongside a decline in function, particularly a reduction in the activity of lysosomal hydrolases [192, 193, 194, 195]. Elevated lysosomal pH has been reported during replicative aging in budding yeast, as well as during organismal aging in *C. elegans* [196, 197]. These alterations may disrupt the acidic environment required for optimal hydrolase activity. Conversely, lifespan‐extending interventions, such as reduced insulin/insulin‐like signaling and DR, restore lysosomal acidity and function, highlighting the causal relationship between lysosome health and longevity [198]. Another study has also reported that the transport of bioactive lipids from lysosomes to the nucleus alters gene expression, thereby modulating lifespan in worms [199].

As integral components of the autophagy–lysosomal degradation system, lysosomes mediate the proteolytic removal and recycling of cellular constituents, encompassing both proteins and organelles. Regardless of whether autophagy occurs via macroautophagy, chaperone‐mediated autophagy, or microautophagy, lysosomes play a central role in all these pathways [200]. Compromised autophagy with age correlates with proteostasis collapse and proteinopathy, while pharmacological or genetic enhancement of autophagy extends lifespan across species [193, 198, 201, 202, 203, 204]. Regulation of different steps of autophagy, including initiation, phagophore formation, cargo loading, and degradation, plays a critical role in modulating longevity and aging [205]. As the role of autophagy in aging has been extensively reviewed elsewhere [201, 202, 206, 207, 208], here we will focus specifically on the lysosome‐associated aspects of this mechanism in the modulation of aging and longevity.

Lysosomal degradative capacity is a critical determinant of autophagy function and efficiency. Although lysosomal degradation is considered the final stage of autophagy, recent studies have shown that lysosomes also actively regulate autophagy, particularly through the mTORC1 kinase complex, which is a key regulator of cell growth [200]. While nutrient availability causes mTORC1 to inhibit autophagosome biogenesis by phosphorylating and inactivating the initiator kinase complex ULK1–ATG13–FIP200, a reduction in food intake or drug‐induced inhibition of mTORC1 leads to activation of autophagy and extends longevity [209, 210]. Remarkably, the accumulation of amino acids in the lysosomal lumen enables mTORC1 recruitment and activation at the lysosomal membrane [211]. Moreover, lysosomal substrate delivery through Microtubule‐associated protein 1A/1B light chain 3‐associated phagocytosis and endocytosis is compromised in aging and age‐related diseases [194]. Additionally, growing evidence indicates that lysosomal protein quality control mechanisms, including ESCRT‐independent membrane repair and autophagy‐related lipid transfer processes, play a critical role in preserving lysosomal integrity and proteostasis in the context of aging and NDs [194, 212, 213].

In addition to its degradative role, the lysosome serves as a storage site for essential ions, including iron and calcium. Lysosomal impairment causes cellular iron deficiency in both yeast and mammals, adversely affecting mitochondrial function, as many enzymes in the mitochondrial respiratory chain require iron [214]. Beyond iron, lysosomal dysfunction also disrupts cellular Ca^2+^signaling [215]. For example, elevated cytosolic calcium resulting from lysosomal malfunction shortens lifespan in yeast [216]. Moreover, the lysosomal proton pump, V‐ATPase, a key component of the autophagy pathway, has been identified as a determinant of longevity in yeast [217]. Loss of lysosomal acidity due to reduced V‐ATPase activity is linked with shortened lifespan, mainly through its detrimental effect on mitochondrial function [218]. Concomitantly, inhibition of V‐ATPase activity, whether by genetic manipulation or pharmacological treatment, results in mitochondrial dysfunction and a shortened lifespan [219].

In addition to its direct involvement in lifespan regulation, lysosomal dysfunction contributes to various age‐related pathologies, including late‐onset NDs. Previous studies have demonstrated that neurons are particularly vulnerable to lysosomal dysfunction [220, 221]. Due to their postmitotic nature, neurons rely heavily on lysosomes to maintain cellular homeostasis, as they cannot dilute cellular waste through cell division. In NDs such as AD, PD, and frontotemporal dementia (FTD), impaired lysosomal function has emerged as a central pathological feature. This impairment arises from multiple factors, including reduced intraluminal acidification, lysosomal enzyme malfunction, and disrupted calcium regulation [193, 204, 222, 223, 224, 225]. Moreover, distinct mutations in presenilin 1 (*PSEN1*), which lead to defects in lysosomal acidification, have been implicated in early‐onset AD in young individuals [226].

Together, preserving lysosomal proteostasis is critical for healthy aging and holds therapeutic potential, not only by counteracting the deleterious effects of aging but also by delaying or alleviating age‐related diseases. Thus, targeting age‐related impairment of lysosomal function, including impaired acidification, decline in enzymatic activity, and compromised autophagic degradation, may represent a beneficial strategy to extend lifespan and prevent age‐related diseases.

## Membraneless organelles

Organelles such as nuclei, mitochondria, ER, lysosomes, and the Golgi are all surrounded by lipid membranes. This compartmentalization is essential for their individual biological functions. In contrast, RNA molecules are rarely enclosed by membranes [227]. Instead, they are typically bound to RNA‐binding proteins (RBP), which can interact via liquid–liquid phase separation (LLPS) [228]. This process gives rise to membraneless organelles, commonly referred to as RNA granules. These include nuclear membraneless organelles, such as the nucleolus (see the section Nucleolus) and paraspeckles (see the section Nuclear speckles), as well as cytoplasmic membraneless organelles.

### Cytoplasmic ribonucleoprotein complexes

Stress granules are a subset of membraneless RNA granules located in the cytoplasm that form in response to cellular stress. They vary in sizes ranging between 0.1 and 2 um^3^ and exhibit a structure of denser regions termed SG‐cores and more dynamic SG‐shells [229, 230]. SGs consist of ribosomal subunits, translation initiation factors, mRNA molecules, and RBPs [231]. Most of these RBPs serve a function in post‐transcriptional control, such as mRNA stability and translational control. Similar to SGs, PBs are also cytoplasmic membraneless RNA granules [7, 232]. They mainly consist of mRNAs and proteins which play a role in translation repression and 5′‐to‐3′ mRNA decay [233]. Both SGs and PBs are highly dynamic, constantly exchanging molecules not only with the cytosol but also with one another. Functionally, they are involved in various mRNA quality control processes [234].

The assembly of SGs in the cytoplasm is linked to the halting of translation and can be triggered by various stress factors, such as heat or oxidative stress, hyperosmolarity, UV‐radiation, and viral infections [235, 236]. Core SG components, such as Ras GTPase‐activating protein‐binding protein 1 (G3BP1) and Ras GTPase‐activating protein‐binding protein 2 (G3BP2), tristetraprolin (TTP), fragile X mental retardation protein (FMRP), ubiquitin‐associated protein 2‐like (UBAP2L), cell cycle‐associated protein‐1 (CAPRIN1), and T‐cell intracellular antigen‐1 (TIA1) serve as nucleating RBPs and form the stable SG core [229, 231]. Subsequently, other proteins with intrinsically disordered regions (IDR) can be further recruited to the SG‐shell. SG‐core proteins have been implicated in age‐dependent protein‐misfolding diseases and NDs. For example, G3BP1 interacts with mutant polyQ‐expanded HTT protein. The knockdown of G3BP1 leads to increased aggregation of mutant HTT and exacerbates disease‐related changes [237]. Additionally, the knockout of G3BP1/2 generally inhibits the formation of SGs under different stress conditions. In turn, this leads to a significant reduction of stress‐induced RNA decay, which highlights the essential role SGs play in RNA and protein quality within the cell [238]. Mutations in the low complexity domain of TIA‐1 alters its phase separation capacity, therefore leading to more persistent SGs and the aggregation of the RBP TAR‐DNA binding protein 43 (TDP‐43), a common hallmark of ALS [239, 240]. In addition, mutations in another SG‐core component, CAPRIN1, have been linked to pathological SG persistence and protein aggregation, which in turn contributes to age‐associated NDs, such as early‐onset ataxia and cognitive decline [241, 242].

Due to their dynamic nature, SGs can disassemble once the stressor is removed. However, under prolonged or chronic stress, such as during aging and associated proteostasis decline, SGs can become aberrant, losing their dynamic properties and persisting in the cytoplasm [243]. Specifically, age‐related cellular environmental and metabolic changes affect SG dynamics. Furthermore, mutations in RBPs that are linked to age‐related NDs also affect SG dynamics. For example, elevated SG formation and impaired clearance have been observed in NDs, such as ALS and FTD [244]. When TDP‐43 harbors disease‐causing mutations, it accumulates and forms abnormal cytosolic inclusions. Similarly, mutations in fused in sarcoma (FUS), another RBP involved in ALS, also lead to its aberrant cytoplasmic mislocalization. Both RBPs have been shown to associate with SGs and are therefore linked to proteostasis decline and progression of NDs [245, 246, 247, 248, 249]. Another protein that mislocalizes from the nucleus to the cytoplasm, leading to the formation of aberrant SGs and contributing to ALS, is the heterogeneous nuclear ribonucleoprotein A2/B1 (hnRNPA2/B1). Knockdown of the ATPase RUVBL2 in rats reduces hnRNPA2/B1‐dependent SG formation, thereby protecting cells from toxic protein aggregation and supporting overall proteostasis [250]. Additionally, the aggregation of specific proteins into SGs during aging may also have beneficial effects. For example, the RBP Human Antigen R (HuR) aggregates into SGs, thereby facilitating osteogenesis through the recruitment of essential mRNAs, such as β‐catenin. Through this recruitment and stabilization of key mRNAs, HuR ensures the correct synthesis of proteins that are critical for bone health during the aging process [251].

Cellular senescence, which contributes to aging, impairs the formation of SGs in kidney cells [252]. Another study in human fibroblasts demonstrated that SGs form in proliferating cells, but not in fully senescent cells, when exposed to stress. The authors further showed that early induction of SG formation reduces the proportion of cells that entered senescence [253]. Senescent cells exhibit altered mTORC1 activity and increased SG formation. SGs can then sequester pro‐apoptotic proteins and, in turn, further modulate mTORC1 activity via negative feedback. This restricts excessive mTORC1 activity that typically leads to apoptosis, thereby enhancing cell survival [254]. Aging also affects the LLPS properties of SGs, altering their biophysical characteristics. These changes can impair RNA metabolism and protein quality control, thereby exacerbating age‐associated cellular decline [255]. Furthermore, a study in *Saccharomyces cerevisiae* showed that mutants lacking both SGs and PBs exhibit increased protein misfolding, further supporting a role of these granules in proteostasis maintenance [256].

In *C. elegans*, SG formation can be induced at the organismal level by conditions, such as DR and starvation, mediated through the AMPK‐eEF2K pathway. This, in turn, regulates proteostasis and contributes to lifespan extension [257]. Numerous studies have demonstrated that aging is associated with a substantial accumulation of detergent‐insoluble proteins in animal tissues [258, 259, 260, 261, 262]. Among them, certain SG components become highly insoluble in aged *C. elegans*. In contrast, long‐lived *daf‐2* mutant animals, a genetic model of reduced insulin/insulin‐like growth factor 1 (IGF‐1) signaling, can efficiently prevent protein aggregation and exhibit extended health span [263]. Notably, PBs also accumulate during aging in somatic tissues of *C. elegans* [264]. This increase correlates with alterations in mRNA metabolism and translational control. Downregulation of mRNA decapping via the EDC‐3 mRNA decapping enzyme enhancer in PBs promotes stress resistance and longevity. Furthermore, PB‐mediated sequestration of specific translation initiation factors increases both during aging and in response to cellular stress in *C. elegans*. Together, these findings highlight a key role of PBs in regulating proteostasis and modulating lifespan.

Taken together, the dynamics and biophysical properties of both SGs and PBs are highly affected during aging and are tightly linked to cellular proteostasis. Conversely, their modulation presents a promising avenue for developing therapeutic approaches to preserve proteostasis and mitigate age‐associated diseases.

### Nucleolus

An important sub‐organelle within the nucleus is the nucleolus, a membraneless substructure responsible for ribosomal RNA synthesis and ribosome subunit assembly, playing a key role in protein synthesis [265]. During aging, increased nucleolar activity correlates with elevated ribosome production, a metabolically demanding process associated with accelerated aging [19]. Conversely, reductions in ribosome biogenesis are linked to increased lifespan in *C. elegans* [266, 267, 268, 269]. Recent studies across multiple model organisms, from yeast to mice, identified that smaller nucleoli are associated with longevity, often linked to decreased ribosome biogenesis and improved cellular homeostasis [270, 271].

In *C. elegans*, long‐lived mutant animals representing distinct longevity paradigms exhibit smaller nucleoli and decreased levels of both rRNA and ribosomal subunits. These effects are mediated by higher activity of NCL‐1 in long‐lived mutants, the worm ortholog of TRIM2/BRAT tumor suppressor, which inhibits production of FIB‐1/fibrillarin, a protein involved in the regulation and maturation of rRNA [271]. Importantly, either overexpression of *ncl‐1* or knockdown of fibrillarin reduces nucleolar size and extends lifespan in wild‐type animals [271]. In addition to ribosome biogenesis, the nucleolus also functions as a protein quality control compartment within the nucleus [272, 273]. Due to the abundance of stress‐sensitive and metastable proteins within the nuclear proteome, highly efficient protein quality control mechanisms are essential to maintain proteostasis. The nucleolus, the largest membraneless subcompartment of the nucleus, contributes to this regulation by acting as a dynamic quality control center. Upon stress, misfolded nuclear proteins transiently accumulate within the liquid‐like granular component of the nucleolus, which serves as a phase‐separated compartment for protein sequestration and recovery [272]. Furthermore, a recent study demonstrated that knockdown of *nol‐56*, which encodes a fibrillarin‐interacting protein, efficiently mitigates disease‐related phenotypes in worm models expressing aggregation‐prone Aβ and polyQ‐expanded proteins, thereby providing a further link between nucleolus and intracellular proteostasis [274].

### Nuclear speckles

After the nucleolus, NS are the second largest nuclear subcompartments. These dynamic ribonucleoprotein complexes reside mainly in DNA‐depleted interchromatin regions. Initially, NSs were considered passive structures involved in the regulation of RNA processing through the temporary storage and assembly of splicing factors. However, recent studies have revealed that NSs actively participate in other aspects of gene expression by promoting transcription of nearby gene loci and facilitating processing of nascent transcripts [275, 276, 277]. Growing evidence implicates NSs' contribution to genome organization and positioning within the nucleus [278].

The core scaffold proteins required for NS nucleation are the IDR‐rich proteins SON and SRRM2, while NS morphology is further shaped by poly‐adenylated RNAs (poly(A)+) [279, 280]. In addition to the core components and RNA‐processing factors, NSs contain RNA polymerase II subunits, transcription factors, small nuclear RNAs (snRNAs), and certain long noncoding RNAs (lncRNAs), such as *MALAT1* [279, 281].

The dynamic properties of NSs are readily modulated under both physiological and stress conditions [282, 283]. Their phase separation dynamics regulate proteostasis by modulating the interaction and recruitment of key transcriptional factors, such as XBP1 [284]. In addition, the spatial proximity of gene loci encoding major heat shock‐induced proteins promotes their expression under stress [282, 285]. NSs also sequester transcripts with retained introns, providing a potential mechanism to downregulate genes that are nonessential under stress conditions [277, 286]. A recent study suggests that NSs contribute to the homeostasis of condensation‐prone proteins by sensing the abundance of IDR‐containing proteins and modulating their levels through sequestration of the corresponding mRNAs [287]. Given that NSs are involved in the regulation of key aspects of RNA and proteostasis, their dysregulation may represent an important contributor to the aging process.

Changes in NS architecture and function can arise from alterations in their molecular composition as well as from changes in post‐translational modifications of their resident proteins [281, 286, 288, 289]. An age‐dependent decrease in NS size, accompanied by reduced SON levels, was reported in aged mice as well as in human fibroblast and brain tissues from patients with AD, while restoring SON expression was sufficient to rescue NS morphology [290]. Moreover, the small molecule pyrvinium pamoate was identified as a potential SON‐dependent NS rejuvenator, as it modulates SON condensation and preserves NS morphology and function in both aging and tauopathy models [290].

Ensuring splicing fidelity is essential for both cellular and organismal homeostasis. However, age‐dependent alterations in splicing patterns have been reported across species, and splicing defects are associated with multiple age‐related pathologies, including NDs [281, 291, 292, 293, 294]. Since NSs harbor regulators of mRNA processing and modification, their dysregulation can influence different aspects of RNA metabolism and eventually proteostasis. For instance, the long isoform of hnRNP D‐like (L‐DL), a splicing regulator dominantly localized to neuronal NSs, is essential for proper splicing of mRNAs encoding synaptic and cytoskeletal proteins. However, a decrease in L‐DL levels alters NS morphology and disrupts splicing patterns of target mRNAs, thereby contributing to cognitive decline in aged mice as well as in AD mouse models [295].

Lamin A, the main constituent of nuclear lamina, is also present in NSs, where it anchors and stabilizes the N6‐methyladenosine (m6A) methylases METTL3 and METTL14 within these nuclear bodies [296, 297]. However, Lamin A deficiency or accumulation of Progerin, as observed in senescent cells, leads to the translocation of METTL3/METTL14 to the cytoplasm, promoting their proteasome‐dependent degradation [297, 298]. This finding is in accordance with recent studies reporting lower METTL3 and global m6A RNA hypomethylation of transcripts involved in synaptic plasticity in aged mice and AD patients [299, 300, 301].

Further evidence implicates NSs in the pathogenesis of age‐related diseases, many of which involve aberrant alternative splicing and protein aggregation. In cellular and mouse models of tauopathies, nuclear tau aggregates localize within NSs, altering their composition and dynamics. Furthermore, numerous NS proteins, including SRRM2 and PNN, mislocalize to cytoplasmic tau aggregates in both mouse and human brains. Polyserine domains within these proteins mediate their interactions with tau aggregates [302, 303]. Additional NS resident proteins have also been implicated in tau‐driven toxicity across various disease models [302, 304]. Toxic repetitive RNA molecules, characteristic of polyQ diseases such as HD and ALS, have been shown to colocalize with NSs [305, 306]. In FTD/ALS caused by mutations in the *C9ORF72* gene, toxic RNAs formed due to the G_4_C_2_ repeat expansion within the first intron of this gene interact with SRRM2, resulting in enlarged and less dynamic NSs. These alterations are accompanied by cytoplasmic SRRM2 accumulation and mRNA mis‐splicing [306]. NS‐driven changes in gene expression have also been reported in several cancer types [299].

While NSs were initially described over a century ago, their multifaceted roles continue to be uncovered with advances in molecular and imaging techniques. Their position at the intersection of RNA and protein metabolism infers their crucial role in maintaining cellular homeostasis, and it is unsurprising that they are indirectly implicated in numerous age‐related diseases. However, further studies will be required to clarify whether NS alterations may play a causative role in aging‐ and disease‐associated dysfunction.

## Conclusion

Maintaining organelle homeostasis during life is a central regulator of cell function and longevity. With age, membrane‐bound organelles such as the nucleus, mitochondria, ER, Golgi, and lysosomes are altered structurally and functionally, which compromises important biological processes, such as genomic stability, energy production, proteostasis, and intracellular signaling. Similarly, membraneless compartments, including SGs and NSs, undergo alterations in different ways, such as changes in phase separation dynamics, impaired RNA metabolism, and loss of proteostasis. Remarkably, cellular organelles do not function alone, but they are tightly interconnected. Therefore, dysfunction in one organelle often propagates to disrupt homeostasis in other organelles. These accumulated impairments promote age‐associated pathologies, such as senescence, chronic inflammation, and neurodegeneration. One example of such organelle contact sites is the ER–mitochondria contact site (ERMCS), which has been well studied in the context of proteostasis [307], whereas many other contact sites remain largely unexplored. Although substantial progress has been made in understanding how individual organelles age and contribute to disease, the mechanisms governing their intercommunication and their role in aging are only beginning to be elucidated. Therefore, it is also essential to put particular emphasis on uncovering the molecular basis of organelle crosstalk and its role in aging and age‐related diseases. Taken together, restoring or maintaining organelle homeostasis and proteostasis emerges as a powerful potential avenue to promote healthy aging.

## Conflict of interest

The authors declare no competing interests.

## Author contributions

All authors contributed to the conceptualization and writing of the review.
